# Insights into the Action Mechanisms of Traditional Chinese Medicine in Osteoarthritis

**DOI:** 10.1155/2017/5190986

**Published:** 2017-01-22

**Authors:** Linfu Li, Haiqing Liu, Weimei Shi, Hai Liu, Jianqiong Yang, Daohua Xu, Hao Huang, Longhuo Wu

**Affiliations:** ^1^College of Pharmacy, Gannan Medical University, Ganzhou 341000, China; ^2^Department of Clinical Research Center, The First Affiliated Hospital of Gannan Medical University, Ganzhou 341000, China; ^3^Department of Pharmacology, Guangdong Medical University, Dongguan 523808, China

## Abstract

Osteoarthritis (OA) is a chronic degenerative joint disease characterized by articular cartilage destruction, synovial inflammation, and osteophyte formation. No effective treatments are available. The current pharmacological medications such as nonsteroidal anti-inflammatory drugs (NSAIDs) and analgesics, accompanied by possible adverse effects, might ameliorate OA symptoms. But they do not arrest the progression of OA. Traditional Chinese medicine (TCM) provides medical value by modification of disease and symptoms in OA. Valuable work on exploring TCM merits for OA patients has been investigated using modern technologies, although the complicated interacting network among the numerous components indicates the uncertainty of target specification. This review will provide an overview of the action mechanism of TCM in the last 5 years, discussing the TCM activities of anti-inflammation, antiapoptosis, antioxidation, anticatabolism, and proliferation in OA. TCM is a proposed medical option for OA treatment.

## 1. Introduction

Osteoarthritis (OA) is a deteriorating joint disease and becomes the major cause of chronic disability, especially for the aged people. The pathological developments in OA include the destruction of cartilage, the inflammation of synovium, the formation of osteophyte, the thickening of subchondral bone, and the degeneration of chondrocytes [1]. The etiological risk for OA involves various factors, including inflammation, oxidative stress, mechanical stress, join injury, age, and other systemic diseases [2]. To date, there is no effective treatment for OA. Unfortunately, the aging society will produce more OA patients. More than 27 million Americans with over $185.5 billion annual medical expenditures have posed a great significant economic burden in USA [3].

The balance driving for anabolic and catabolic activities in articular cartilage is controlled by signaling pathways. However, this balance is tilted towards joint destruction. Various signaling pathways are involved in OA to impair the homeostasis of articular cartilage. These include Wnt/*β*-catenin pathway [4], the nuclear factor-*κ*B (NF-*κ*B) pathway [5], the p38, ERK1/2 and JNK MAP kinase [6], and the PI-3 kinase- (PI3K-) Akt pathway [6], which have been comprehensively reviewed [7, 8]. Although the inflammation of synovial membrane and the degeneration of articular cartilage are the two major features of OA, the underlying mechanisms responsible for joint destruction are still unclear. With Lack of detailed and clear information about the pathology of OA, it is quite difficult to develop effective strategies for OA management.

Currently, the goals for managing OA are to ameliorate the painful symptoms, minimize disability, and enhance the life quality. The treatment options recommended in clinic are involving surgery, the existing pharmacological medication, or nonpharmacological therapies, such as exercises and weigh loss [9]. The pharmacological intervention in clinical practice mainly consists of nonsteroidal anti-inflammatory drugs (NSAIDs), analgesics, hyaluronic acid, and corticosteroids [10]. However, these pharmacological medications do not meet patients' expectancy and even cause serious side effects. Traditional medicine including some nutraceuticals acts as the promising alternative medicine as a treatment option for OA and might be easily adopted by clinicians and patients (Table 1). Evidences that support physiological and functional value of nutraceuticals for OA have been comprehensively reviewed by Henrotin et al. [11] and Leong et al. [12]. In this review, we will summarize the action features of TCM and their components that positively contribute to ameliorate the pathological changes in cartilage and chondrocytes.

## 2. Anti-Inflammatory Activity of TCM

**Figure 1:**
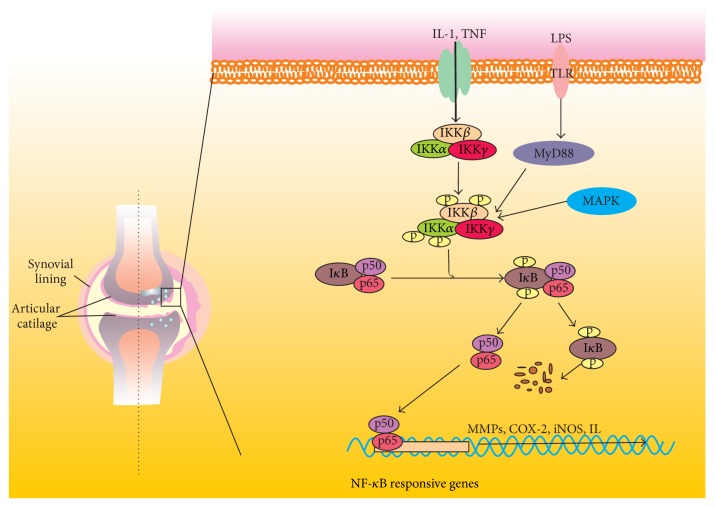
The activity of NF-*κ*B signaling in OA chondrocytes. NF-*κ*B signaling may be activated by many detrimental stimuli, such as IL-1, TNF, and LPS, leading to upregulation of MMPs, COX-2, iNOS, and IL. Crosstalk with MAPK also promotes NF-*κ*B signaling.

Inflammation is closely related not only to symptoms and signs but also to progressive cartilage loss in OA. The low-grade inflammatory response greatly triggers the development of OA. IL-1*β* and TNF*α* are the two major catabolic inflammatory cytokines to drive the degradation of cartilage. The proinflammatory cytokines implicated in OA can activate NF-*κ*B signaling (Figure 1), which in turn controls the expression of these cytokines. This makes a vicious cycle [13]. In addition, NF-*κ*B signaling interacts with other pathways to deteriorate the degeneration of cartilage.

Inflammatory cytokines induce upregulation of the catabolic activity of matrix metalloproteinases (MMPs) through activation of NF-*κ*B signaling pathway. Aucubin, a natural occurring compound from* Aucuba japonica* and* Eucommia ulmoides*, has been reported to exhibit anti-inflammatory activity by inhibiting the degradation and phosphorylation of I*κ*B and blocking the translocation of NF-*κ*B p65. In IL-1*β*-stimulated chondrocytes, aucubin downregulates the increased expression of MMPs, COX-2, and iNOS and the enhanced production of NO [14]. Honokiol, extracted from* Magnolia officinalis*, possesses anti-inflammatory and antioxidative activities. Honokiol can significantly inhibit IL-1*β*-induced upregulation of IKK/I*κ*B*α*/NF-*κ*B signaling pathway, leading to suppression of the expression of iNOS, COX-2, NO, PGE_2_, IL-6, and MMP-13 [15]. However, honokiol administration might induce embryo-fetal development toxicity [16]. Heat shock protein 90*β* (Hsp90*β*), a member of Hsp90 family of protein chaperones, has been reported to be related to inflammation in rat arthritis. Celastrol is known to suppress immune activation. Celastrol is also an inhibitor of Hsp90*β* to significantly downregulate the expression of MMP-1, MMP-3, MMP-13, iNOS-2, and COX-2 induced by IL-1*β* [17].

Black pepper* (Piper nigrum)*, a spice in general diets, is often used as a traditional medicine. Piperine is an active phenolic constituent and has antiarthritis and anti-inflammatory effects on IL-1*β*-induced fibroblast-like synoviocytes to benefit some inflammatory diseases that are accompanied by pain, such as OA and RA. Reports show that piperine significantly inhibits IL-1*β*-induced activation of NF-*κ*B signaling, leading to downregulation of COX-2, PGE2, iNOS, NO, and MMPs in OA [18]. Bavachin, isolated from* Psoralea corylifolia*, is one of medicinal phytoestrogens that exhibit anti-inflammatory activity. Bavachin potently protects cartilage from inflammation-mediated damage through decreasing IL-1*β*-induced nuclear translocation of p65 and p50 and degradation of I*κ*B*α* but not activator protein-1 (AP-1) DNA-binding activity [19]. Astragalin, a main constituent isolated from* Rosa agrestis*, exhibits anti-inflammatory effects in OA by activating PPAR*γ*, leading to inhibition of IL-1*β*-induced NF-*κ*B and MAPK activation and reduction of NO and PGE_2_ production, as well as iNOS and COX-2 expression [20]. Delphinidin is an anthocyanidin compound isolated from pigmented fruits and vegetables and possesses antioxidative and anti-inflammatory activities. It is found that delphinidin can inhibit IL-1*β*-induced NF-*κ*B signaling through modulating the phosphorylation of IRAK-1^Ser376^, resulting in suppression of COX-2/PGE_2_ expression in OA chondrocytes [21]. Tetrandrine, a main bisbenzylisoquinoline alkaloid extracted from* Stephania tetrandra *S. Moore, inhibits the expression of IL-6, IL-1*β*, TNF*α*, PGE_2_, and NO via blocking I*κ*B*α* and NF-*κ*B p65 phosphorylation in LPS-induced ATDC5 cells [22]. Possibly, tetrandrine administration can cause pulmonary toxicity and metabolic disorder [23].

Resveratrol (trans-3,4-trihydroxystilbene), a natural phytoalexin isolated from cranberries, peanuts, the skin of red grapes, and the root of the weed* Polygonum cuspidatum*, possesses anti-inflammatory, antitumor, and antioxidative activities. Resveratrol is a natural SIRT1 activator to exhibit an anti-inflammatory activity via inhibition of NF-*κ*B. The mechanism might be that resveratrol-activated SIRT1 suppresses not only the nuclear translocation of NF-*κ*B but also the acetylation of p65, leading to downregulation of iNOS expression [24]. Resveratrol has been confirmed to inhibit the expression of COX-2 and iNOS, IL-1*β*, and TNF*α* via downregulation of NF-*κ*B signaling pathway [25]. It also increases the production of type II collagen at mRNA and protein levels in the superficial and middle zones, but much less in the deep zone in cartilage [26].

Catechins, isolated from* Camellia sinensis*, are the main effective compounds of tea polyphenols. (−)-Epigallocatechin-3-gallate (EGCG), one of the most abundant catechins, is the great nutraceutical tea polyphenol in OA, which has been reviewed by Ahmed [27]. EGCG can significantly suppress the expression of COX-2, PGE2, and IL-8 dose-dependently in IL-1*β*-induced human synovial fibroblasts via reducing the phosphorylation of IKK*β* [28]. Calcium crystals formation is considered to be a factor implicated in synovial inflammation. Studies show that EGCG exhibits chondroprotective activity through reducing the inflammatory response induced by calcium pyrophosphate crystals in vitro [29]. EGCG might be the potential candidate for OA treatment by targeting epithelial neutrophil activating peptide-78 (ENA-78), granulocyte macrophage colony stimulation factor (GM-CSF), growth-related oncogene (GRO), GRO-*α*, IL-6, IL-8, monocyte chemotactic protein-1 (MCP-1), MCP-3, macrophage inflammatory protein-1beta (MIP-1*β*), granulocyte chemotactic protein-2 (GCP-2), MIP-3alpha, interferon-gamma-inducible protein-10 (IP-10), nucleosome assembly protein-2 (NAP-2), and leukemia inhibitory factor (LIF) through downregulation of NF-*κ*B and JNK-MAPK signaling pathways [30]. In the EGCG-treated (intraperitoneal injection) mice, articular cartilage shows downregulation of MMP-1, MMP-3, MMP-8, MMP-13, ADAMTS-5, IL-1*β*, and TNF*α* mRNA and upregulation of CBP/p300 interacting transactivator with ED-rich tail 2 (CITED2), which suppresses MMPs transcription [31].

Connective tissue growth factor (CTGF; also known as CCN2), an inflammatory cytokine, is highly expressed in OA. CCN2 can increase the production of IL-1*β* in osteoarthritis synovial fibroblasts through generation of *α*_v_*β*_3_/*α*_v_*β*_5_ integrin-dependent ROS and subsequent activation of signal-regulating kinase 1 (ASK1), p38/JNK, and NF-*κ*B signaling pathways. Berberine, an isoquinoline alkaloid isolated from the* Rhizoma coptidis*, effectively prevents cartilage degradation by antagonizing the effect of CNN2 [32]. In addition, it has been demonstrated that downregulation of *α*_v_*β*_3_ integrin is also modulated by berberine through posing the inhibitory effect on PKC*δ*, c-Src, and NF-*κ*B signaling pathways in human chondrosarcoma [33]. Recently, berberine at a relative dose can stimulate the hermetic dose response from clinical applications, particularly in the management of cancer [34]. Toll-like receptor 4 (TLR4) elicits inflammatory responses to develop antigen-specific adaptive immune responses. TLR4 is not only expressed by immune cells, but also expressed by nonprofessional antigen presenting cells (such as cartilage chondrocytes). It has been demonstrated that the expression of TLR4 is increased in chondrocytes from OA patients, indicating that TLR4 might be involved in the OA development [35]. Study shows that resveratrol significantly suppresses the activation of NF-*κ*B signaling induced by TLR4 in RAW264.7 cells [36]. In human chondrocytes, resveratrol can remarkably suppress the upregulation of TLR4 and the downstream target MyD88 and TRAF6 induced by IL-1*β* [37].

On the other hand, Withaferin A, isolated from* Withania somnifera*, can induce the loss of type II collagen, reactive oxygen species, and inflammation in rabbit articular chondrocytes. Evidences show that Withaferin A stimulates downregulation of type II collagen and upregulation of COX-2 through activation of PI3K/Akt, p38, and JNK signaling pathways [38].

## 3. Antiapoptosis and Antioxidative Activities of TCM

Chondrocyte apoptosis is closely related to the progression of OA. Chondrocytes apoptosis can be stimulated by reactive oxygen species (ROS), which can be produced by hydrogen peroxide (H_2_O_2_). Proinflammatory cytokines can enhance the level of ROS via activation of NF-*κ*B signaling pathway. ROS promotes the mitochondrial permeability transition (MPT), resulting in release of cytochome* c* into the cytoplasm. Ginsenoside Rb1, a pivotal component of ginseng, exhibits the activity of inhibiting H_2_O_2_-induced MPT, the expression of caspase-3, and the imbalance of Bcl-xL/Bax ratio [39]. Rb1 inhibits H_2_O_2_-elicited NO and iNOS production and suppresses the proinflammatory cytokines IL-1*β* and TNF*α* expression [40, 41]. Rg1, Rg3, Rg5, Rk1, Rf, Rd, Rc, and F4 are saponins and have similar chondroprotective activity [42–44]. They inhibit IL-1*β*-induced chondrocytes apoptosis by enhancing Bcl-2/Bax ratio and inhibiting the cytochome* c *release. Also, they promote the expression of TIMP-1 and inhibit the expression of MMP-13 through downregulating the PI3K/Akt signaling pathway [42]. Bax, Bad, p53, COX-2, and p65 are closely related to chondrocytes apoptosis. Ro, another oleanolic acid-type ginsenoside, suppresses cell apoptosis by inhibiting the levels of Bax and Bad, decreasing phosphorylation of p53, and promoting the expression of Bcl-xL and PCNA. Moreover, Ro inhibits the phosphorylation of NF-*κ*B p65 induced by IL-1*β* [45].

Proinflammatory cytokines such as IL-1*β* and TNF-*α* (IT) can stimulate the caspase signaling to induce cell apoptosis. Baicalein, a main active compound isolated from* Scutellaria baicalensis *Georgi, exhibits antiapoptotic activity by decreasing NO production and inhibiting the caspase cascade activation [46]. NO donor sodium nitroprusside (SNP) is commonly used as an inducer to trigger apoptosis. Berberine has been reported to reverse SNP-induced cytoskeletal remodeling and chondrocytes apoptosis with downregulation of the expression of iNOS and caspase-3 and upregulation of Bcl/Bax ratio and production of type II collage. These are accompanied by activation of AMPK phosphorylation and suppression of p38 MAPK phosphorylation [47]. Because of poor aqueous solubility, berberine has a low bioavailability and short biological half-life. Berberine-loaded chitosan nanoparticles are designed. They confer stronger activity of antiapoptosis in OA through qRT-PCR, Western blot, and immunohistochemical analyses of caspase-3, Bcl-2, and Bax expression [48].

NO and ROS are responsible for SNP-induced chondrocytes apoptosis through an intrinsic apoptosis pathway. Resveratrol has been demonstrated to scavenge SNP-induced ROS, instead of NO, to remarkably prevent chondrocytes apoptosis [49]. Using atomic force microscopy (AFM), resveratrol potently prevent SNP-induced chondrocytes changes, which include shrunk, round, lamellipodia contraction, and aggregation of the cytoskeleton, decrease in adherent junctions among cells, and decrease in the expression of cytoskeletal proteins [50]. Duhuo Jisheng decoction, a Chinese traditional herbal formula, increases Bcl-2 expression, whereas it decreases the expression of Bax, caspase-3, and caspase-9 induced by SNP to inhibit chondrocytes apoptosis in a mitochondrial dependent manner [51]. Fuyuan Decoction (FYD) has been demonstrated to decrease I*κ*B*α* degradation and reduce the content of p65 in the nucleus induced by IL-1*β*, resulting in suppression of iNOS and NO expression in SW1353 cells [52]. Tetramethylpyrazine, a main active component isolated from* Ligusticum wallichii Franchat*, inhibits the chondrocytes apoptosis through downregulation of ROS and caspase-3 expression and maintenance of mitochondrial membrane potential [53].

Chondrocytes apoptosis can also be triggered by endoplasmic reticulum (ER) stress, which is caused by the accumulation of unfolded or misfolded proteins. In tunicamycin- (TM-) induced chondrocytes, Bushen Zhuangjin Decoction decreases the mRNA and protein expression of BIP, ATF4, CHOP, caspase-9, caspase-3, and Bax and increases the expression of XBP1 and Bcl-2 [54]. Our research group shows that 5,7,3′,4′-tetramethoxyflavone (TMF), an active compound isolated from* Murraya exotica *(L.), can inhibit chondrocytes apoptosis through downregulation of the increased EP/cAMP/PKA signaling pathway and *β*-catenin signaling pathway induced by PGE_2_ [55]. In addition, TMF also suppresses TM-induced ER stress by downregulating the three membrane proteins PERK, ATF6, and IRE1 signaling pathways [56]. Nicotine activates the nicotinic acetylcholine receptors (nAChR) to resist cell apoptosis and promote cell proliferation [57]. It has been demonstrated that nicotine blocks cell apoptosis by neutralizing IL-1*β*-induced downregulation of PI3K/Akt signaling pathway, including PI3K/Akt/Bcl-2 signaling in chondrocytes [58].

## 4. Anticatabolic Activity of TCM

Articular cartilage extracellular matrix (ECM) plays a crucial role in regulating chondrocyte metabolism and functions. ECM is constituted primarily by type II collagen and large networks of proteoglycans (PGs) that contain glycosaminoglycan (GAG), hyaluronic acid (HA), and chondroitin sulfate (CS) [59]. Destruction of the articular cartilage in OA might be due to the combination of increased degradation of ECM, decreased production of ECM, and chondrocyte death. MMPs and a disintegrin and metalloproteinase with thrombospondin motifs (ADAMTS) play the pivotal roles in the overactive catabolic destruction of cartilage.

Sanmiao formula, a Chinese traditional medicinal prescription since the Ming Dynasty, exhibits protective activity for cartilage through decreasing the expression of MMP-3 and ADAMTS-4 and augmenting the expression of TIMP-1 and TIMP-3. In addition, it also inhibits the expression of IL-1*β* and TNF*α*, which are associated with activation of NF-*κ*B signaling regulating the activity of MMPs [60]. Biochanin A, an isoflavone isolated from red clover, exhibits antiallergic, anticancer, and anti-inflammatory activity. Investigation indicates that biochanin A inhibits the increased expression of MMPs and enhances the decreased TIMP-1 expression induced by IL-1*β* in chondrocytes, which might be associated with attenuation of NF-*κ*B signaling [61]. Crocin is a main active compound isolated from* Crocus sativus* L. (saffron) and does not harm live and other organ's function within the pharmacological doses [62]. It has been showed that Crocin ameliorates cartilage degeneration and decreases the expression of MMP-1, MMP-3, and MMP-13 through inhibition of NF-*κ*B signaling [63]. Juanbi capsule is a Chinese medicine for preventing OA. It has been found that Juanbi capsule in vivo can effectively protect cartilage and significantly decrease serum MMP-2 and MMP-9 levels [64]. Sinomenine, an alkaloid isolated from* Sinomenium acutum*, can dose-dependently inhibit the release of glycosaminoglycans (GAG) and the expression of MMP-13 and caspase 3 and enhance the activity of TIMP-1, leading to prevention of DNA fragment and cell apoptosis in chondrocytes [65]. Monotropein, an iridoids glycoside isolated from the roots of* Morinda officinalis How*, inhibits IL-1*β*-induced upregulation of MMP-3 and MMP-13 and promotes the expression of COL2A1 [66].

Nicotine promotes protein synthesis and modulates the activity of MMP-13 and TIMP-1 to maintain ECM balance in cartilage through upregulating PI3K/Akt/p70S6K/S6 signaling pathway [67]. In an experimental rat OA model, berberine decreases the expression of MMP-1, MMP-3, and MMP-13 whereas it increases TIMP-1 at the mRNA and protein levels [68]. The underlying mechanism might be associated with activation of Akt and p70S6K/S6 signaling pathways, which are involved in the chondroprotective activity of berberine in maintenance of cell survival and promotion of matrix production [58].* Phellodendron amurense* is widely used as an anti-inflammatory and immunostimulatory medicine. Evidence shows that* Phellodendron amurense* protects joint cartilage from induction of IL-1*α* through inhibiting the release of proteoglycan and the degradation of type II collagen, decreasing the activities of aggrecanases, MMP, p-ERK1/2, JNK, and p38 MAPK signaling, and increasing the activity of TIMP-1 [69]. Gentiopicroside, a main effective component of secoiridoid glycosides isolated from* Gentiana macrophylla *Pall, inhibits the phosphorylation of p38, ERK, and JNK and the expression of MMPs induced by IL-1*β*. In addition, it enhances the production of type II collagen [70]. Baicalein reduces the expression of MMP-3 and MMP-13 and enhances the production of GAG and type II collagen [46]. These might be associated with downregulation of phosphorylation of p38 and ERK but not of JNK [71]. Tetramethylpyrazine has been showed to decrease the degradation of GAG and the expression of MMP-3, MMP-13, COX-2, iNOS, and type X collagen and increase the expression of TIMP-1 and type II collagen [53, 72].

Astaxanthin is a natural red carotenoid pigment and possesses antioxidative and anti-inflammatory activity in cartilage. Investigation indicates that astaxanthin can decrease the expression of MMP-1, MMP-3, and MMP-13, inhibit the phosphorylation of p38 MAPK and ERK1/2, and block the degradation of I*κ*B*α* in IL-1*β*-induced chondrocytes [73]. Bee venom is a natural ingredient produced by the honey bee* (Apis mellifera)*. It has been demonstrated that bee venom can inhibit the TNF*α*-induced increased expression of MMP-1 and MMP-8 through downregulation of NF-*κ*B and AP-1 signaling pathways. In addition, bee venom also can suppress the phosphorylation of Akt, JNK, and ERK1/2 induced by TNF*α*. But it does not affect p38 phosphorylation [74]. Schisandrae Fructus (SF) is the dried fruit of* Schisandra chinensis* (Turcz.) Baill. (Magnoliaceae). The ethanol extract of SF can significantly exhibit chondroprotective features and attenuate the expression of MMP-1, MMP-3, MMP-13, COX-2, and iNOS through suppression of NF-*κ*B signaling and p38, ERK1/2, and JNK phosphorylation [75]. Morin (3,5,7,2′,4′-pentahydroxyflavone), a flavonoid extracted from the Moraceae family, possesses antioxidative, anti-inflammatory, and antitumor activities. Studies show that morin inhibits IL-1*β*-induced phosphorylation of p38 and ERK1/2, decreases the expression of MMP-3 and MMP-13, and increases TIMP-1 expression [76]. Investigation on the effect of an aqueous extract of* Eucommia ulmoides* on the articular cartilage has been carried out. The results show that a lower Mankin's grade induced by* Eucommia* is involved in histopathological examination. The levels of MMP-1, MMP-3, and MMP-13 in the serum and synovial fluid are negatively controlled by* Eucommia* [77]. These might be associated with downregulation of PI3K/Akt signaling pathway [78].

**Figure 2:**
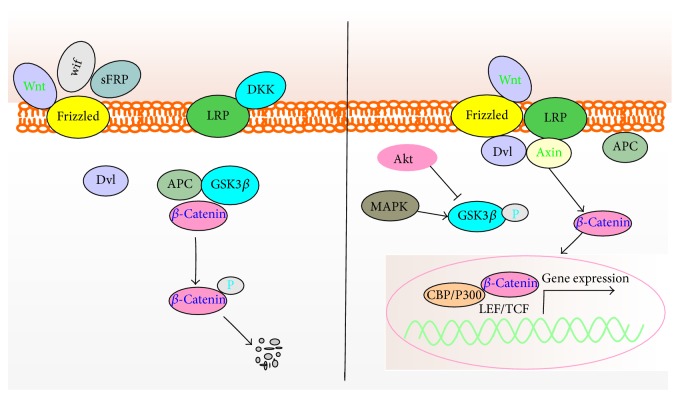
The Wnt/*β*-catenin signaling in OA chondrocytes. Inhibitory phosphorylation of GSK-3*β* promotes the stability of *β*-catenin, which enters the nucleus for transcription.

Icariin, isolated from* Epimedium pubescens*, is a pivotal effective compound to be related to multitherapeutic activities. Icariin exhibits chondroprotective effect and promotes the synthesis of ECM through upregulation of SOX9, type II collagen, and aggrecan in chondrocytes [79], which might be related to inhibition of NF-*κ*B signaling, leading to the suppression of the increased expression of MMP-13 induced by IL-1*β* in vivo and in vitro [80]. MAPK and Wnt/*β*-catenin signaling are also involved in this modulation. Icariin is positively related to the decreased phosphorylations of p38, JNK, and *β*-catenin. This suggests that icariin exerts a promising chondrogenic effect on cartilage tissue engineering [81]. Rb1 upregulates the expression of chondrogenic genes type II collagen and SOX9. Whereas it downregulates the ECM catabolic factors MMP-1 and MMP-13 [40, 41]. Additionally, MMP-13 is positively associated with Notch signaling, which can be suppressed by Rb1 [82]. Pinocembrin, a flavonoid extracted from propolis, inhibits the nuclear translocation of p65 and phosphorylation and degradation of I*κ*B*α* induced by TNF*α* in human chondrocytes, leading to suppression of MMP-3 and MMP-13 expression [83]. Ferulic acid is a natural occurring product from* Angelica sinensis* (Oliv.) Diels. Biological activity investigation shows that ferulic acid significantly downregulates the hydrogen peroxide-induced IL-1*β*, TNF-*α*, MMP-1, and MMP-13 expression and upregulates SOX9 gene expression [84]. Tetrandrine exhibits chondroprotective activity in vivo and in vitro through inhibiting the increased expression of *β*-catenin signaling (Figure 2) and MMPs induced by IL-1*β* and enhancing the expression of TMP-1 [85]. Qi-Fang-Xi-Bi-Granules (QFXBG) is TCM granules used for the treatment of OA. Fangchinoline and tetrandrine are used as the markers for quality control of QFXBG by HPLC in commercial [86].

In OA, advanced glycation end products (AGEs) are upregulated in cartilage. The accumulation of AGEs can decrease synthesis of proteoglycan and collagen and increase expression of MMPs. These are related to activation of NF-*κ*B and AP-1 signaling pathways [87]. It has been demonstrated that resveratrol can protect cartilage and suppress AGEs-induced expression of COX-2, iNOS, and MMP-13 through downregulating IKK-I*κ*B*α*-NF-*κ*B and JNK/ERK-AP-1 signaling pathways [88]. Resveratrol can synergize curcumin to stimulate the MAPK signaling pathway in human chondrocytes in vitro [89]. Curcumin, a highly pleiotropic molecule extracted from the rhizomes of* Curcuma longa*, presents great potential and excellent safety profile for treating OA, although it has a low solubility and poor bioavailability. The nutraceutical effects of curcumin in OA development have been comprehensively reviewed [90], and here we are not ready to discuss it too much.

From the conventional wisdom of converting skin/hide matrix into leather, it has been hypothesized that polyphenols might cross-link with type II collagen through hydrophobic association and hydrogen bonding. Investigations indicate that polyphenols (combination of EGCG, quercetin, catechin, and tannic acid) administrated by intra-articular injection can bind to collagen in bovine articular cartilage explants, which results in stabilization of cartilage collagen and resistance to degradation by collagenases [91]. To study the effects of polyphenols on anti-inflammation and anticatabolism in OA, Horcajada et al. found that rutin and the mixture of rutin/curcumin could downregulate the expression of Coll2-1, the combination of rutin/curcumin could decrease Fib3-1 and Fib3-2 expression, and Coll2-1NO2 could be significantly downregulated by rutin, curcumin, and oleuropein [92].

The effect of the saponin fraction from* Clematis chinensis* Osbeck roots (SFC) on cartilage in a rat model induced by intra-articular injection of monosodium iodoacetate (MIA) has been investigated. The results show that SFC effectively ameliorates joint destruction and cartilage damage induced by MIA or SNP through blocking the degradation of ECM and preventing chondrocytes injury [93].

## 5. Proliferative Activity of TCM

Cyclin D1, CDK4, and CDK6 are the factors which interact with each other and form a complex to promote the cell cycle.* Achyranthes bidentata* polysaccharides are found in chondrocytes in vitro to induce G1/S cell cycle transition and type II collagen expression by upregulating the expression of CDK4, CDK6, and Cyclin D1, which are related to the Wnt/*β*-catenin signaling pathway [94]. Bushen Zhuangjin Decoction [95] and Duhuo Jisheng Decoction [96] are also found to increase the expression of CDK4, CDK6, and Cyclin D1 and accelerate G1/S cell cycle transition, whereas they decrease the expression of p21.* Bauhinia championi* (Benth.) Benth. polysaccharides (BCBPs) also have been found to promote G1/S cell cycle transition and induce chondrocytes proliferation. BCBPs are composed of at least seven monosaccharides, such as rhamnose, D-(+) glucuronic acid, D-mannose, D-(+) galacturonic acid, galactose, D-glucose, and arabinose. It has been demonstrated that BCBPs can activate the expression of Wnt/*β*-catenin signaling pathway (Figure 2) via upregulating the mRNA and protein expression of Frizzled-2, Wnt-4, *β*-catenin, and Cyclin D1 and downregulating the expression of GSK-3*β* [97].

TGF*β* and SOX9 signaling pathways are of importance for the pathophysiology of joint cartilage. Generally, TGF*β* predominantly phosphorylates Smad2/3 through ALK5, leading to forming a complex with Smad4 and translocating into the nucleus to regulate the target genes expression such as aggrecan and type II collagen. Fuyuan Decoction, a Chinese traditional herbal formula, can promote chondrocytes proliferation and reverse the decreased phosphorylation of Smad2/3 and the decreased expression of COL2A1 and SOX9 induced by IL-1*β* [98].

Protocatechuic acid, one of polyphenolic compounds extracted from green tea and catechins, possesses analgesic and anti-inflammatory activity. Protocatechuic acid can promote the proliferation of rabbit chondrocytes and maintain cell phenotype through enhancing the synthesis of ECM and the expression of aggrecan, type II collagen, and SOX9 [99]. Psoralen, an effective component isolated from* Fructus Psoraleae*, dose-dependently enhances the production of GAG and type II collagen and increases the expression of SOX9 [100]. On the other hand, high dose administration of psoralen may induce writhing, lassitude, and hypoactivity. But the significant toxic side effects of psoralen on bone marrow or other organs such as heart, lung, liver, and spleen are not seen [101].

However, among a vast number of chemicals related to cigarette smoking, nicotine is one of the leading candidates for causing delayed chondrogenesis. The possible mechanism might be that nicotine increases fetal blood corticosterone, inhibits matrix synthesized by growth plate chondrocytes, and downregulates the expression of IGF-1 signaling in chondrocytes, which promotes longitudinal growth by activating the synthesis of ECM [102]. Additionally, the adverse effects of nicotine have been reported to affect many systems, including neuromuscular, cardiovascular, neurological, immunological, respiratory, and gastrointestinal [103].

## 6. Miscellaneous

Shu-Jing-Huo-Xie-Tang (mainly composed of* Cortex Eucommiae*), Du-Huo-Ji-Sheng-Tang* (Radix Angelicae Pubescentis)*, and Shao-Yao-Gan-Cao-Tang* (Radix Dipsaci)* are the most frequency of prescriptions of TCM for diseases of the musculoskeletal system and connective tissue in clinic [104]. Du-Huo-Ji-Sheng-Tang has been demonstrated to show the therapeutic effects on OA in vivo to protect joint tissue through inhibiting the mRNA expression of VEGF and HIF-1*α* [105]. Guilu Erxian Jiao (a Chinese traditional formula) is commonly used remedy for knee OA. From the clinical trial, it has been found that Guilu Erxian Jiao can significantly increase muscle strength and decrease joint pain and Lequesne index scores in OA patients after 12 weeks' administration [106].

Tougu Xiaotong capsule formula, a TCM, promotes cell proliferation, reduces cell mortality, and protects cell from inflammatory cytokine, like TNF*α*, and injury [107]. Yaotongning Capsule (YTNC), a TCM formula, is used for OA treatment in clinic and has a potent activity to prevent OA chondrocytes from degeneration. To improve the quality controllability and safety and achieve maximal therapeutic efficacy, the active fractions of YTNC are reshuttled according to the formulation of YTNC and the concept of combinational chemistry. The results show that the formula can be further simplified according to the reasonable combination of alkaloids, flavonoids, and 50% of saponins from* Glycyrrhiza uralensis*, which is an important minister drug in YTNC [108].

*Aconitum carmichaeli *Debx offers various therapeutic activities, but they are accompanied by acute toxicity due to the aconitine constituents. It can be detoxicated by reducing the aconitine contents. Detoxicated* Aconitum carmichaelii *Debx shows chondroprotective activity in preventing cartilage degeneration, decreasing the bone density and the Mankin score, and promoting chondrocytes proliferation [109].

HIF-2*α* is recently verified to exhibit a crucial role in regulating cartilage destruction by directly inducing expression of catabolic factors, including MMPs, ADAMTS4, iNOS, and prostaglandin-endoperoxide synthase-2 (PTGS2). However, HIF-2*α* is regulated by SIRT1. The injection administration of resveratrol can significantly upregulate SIRT1 expression and downregulate the HIF-2*α* expression, leading to suppression of iNOS and MMP-13 expression [110]. Our research group shows that* Murraya exotica* (L.) can dose-dependently downregulate the mRNA and protein expression of *β*-catenin and COX-2, inhibit the levels of TNF*α* and IL-*β* in synovial fluids, and decrease the chondrocytes apoptosis [111].

## 7. Concluding Marks

This is a systemic review investigating the action mechanisms of TCM and their components regarding activities of anti-inflammation, antiapoptosis, antioxidation, anticatabolism, and proliferation in OA. Although the preclinical and clinical trials of TCM are on the initial step, the data collected indicate a promising beneficial effect on OA. Valuable work has been investigated to further explore the clinical merits of TCM (Table 1). They are primarily focusing on inhibition of proinflammatory cytokines activities, suppression of ROS-induced mitochondrial signaling, downregulation of NF-*κ*B, MAPK, and Wnt/*β*-catenin signaling, inhibition of MMPs and ADAMTS activities, and increase of anabolic activity. However, the action mechanisms of TCM in OA have not yet been fully formulated or still under investigation. Under the light of the theory of TCM, the whole combination of different medicines is critically important for its clinical effects. The biological effects of TCM are more than the sum of activities produced by the individual. In addition, because of a vast number of components and the positively or negatively interacting network among these effective compounds, TCM is currently limited by the uncertainty of target specification.

## Figures and Tables

**Table 1:** Summary of the biological effects of TCM on OA.

List	Compounds	Source or TCM	Cell lines	Biological effects of TCM	Ref.
(1)	Astaxanthin	Marine animals and plants	Human OA chondrocytes	↓MMP-1, ↓MMP-3, ↓MMP-13, ↓phosphorylation of p38 MAPK and ERK1/2, ↓NF-*κ*B signaling	[72]
(2)	Astragalin	*Rosa agrestis*	Human OA chondrocytes	↓iNOS, ↓COX-2, ↓NO, ↓PGE_2_, ↓NF-*κ*B signaling, ↓MAPK signaling, ↑PPAR*γ*	[20]
(3)	Aucubin	*Aucuba japonica* *Eucommia ulmoides*	Rat chondrocyte	↓MMPs, ↓COX-2, ↓iNOS, ↓NO ↓NF-*κ*B signaling	[14]
(4)	Baicalein	*Scutellaria baicalensis*	Rat chondrocyte Human OA chondrocytes	↓caspase signaling, ↓NO, ↓MMP-3, ↓MMP-13, ↑GAG, ↑type II collagen, ↓phosphorylation of p38 and ERK	[45, 46, 70]
(5)	Bavachin	*Psoralea corylifolia*	CHON-002	↓NF-*κ*B signaling	[19]
(6)	Bee venom	*Apis mellifera*	Mouse chondrocytes	↓MMP-1, ↓MMP-8, ↓NF-*κ*B and AP-1 pathways, ↓phosphorylation of Akt, JNK, and ERK1/2	[73]
(7)	Berberine	*Rhizoma coptidis*	SW1353, Rat chondrocyte Rat in vivo	↑Bcl-xL/Bax ratio, ↓CNN2, ↓*α*_v_*β*_3_ integrin, ↓PKC*δ*, ↓c-Src, ↓iNOS, ↓caspase-3, ↓NO, ↓NF-*κ*B signaling, ↓MAPK signaling, ↑AMPK signaling, ↓MMP-1, ↓MMP-3, ↓MMP-13, ↑TIMP-1, ↑Akt/p70S6K/S6	[33, 46, 47, 57, 67]
(8)	Biochanin A	Red clover	rabbit chondrocyte	↓MMPs, ↑TIMP-1, ↓NF-*κ*B signaling	[60]
(9)	Celastrol	*Tripterygium wilfordii *Hook F.	Human OA chondrocytes	↓MMP-1, ↓MMP-3, ↓MMP-13, ↓iNOS-2, ↓COX-2	[17]
(10)	Crocin	*Crocus sativus* L.	Rabbit in vivo and in vitro	↓MMP-1, ↓MMP-3, ↓MMP-13, ↓NF-*κ*B signaling	[62]
(11)	Delphinidin	Pigmented fruits, vegetables	Human OA chondrocytes	↓COX-2, ↓PGE_2_, ↓NF-*κ*B signaling, ↓phosphorylation of IRAK-1^Ser376^	[21]
(12)	EGCG	*Camellia sinensis*	C57BL/6 mice in vivo, human synovial fibroblasts	↓COX-2, ↓PGE2, ↓IL-8, ↓TNF*α*, ↓IL-1*β*, ↓MMP-1, ↓MMP-3, ↓MMP-8, ↓MMP-13, ↓ADAMTS-5, ↓NF-*κ*B signaling, ↓JNK-MAPK signaling, ↑CITED2	[27–31]
(13)	Ferulic acid	*Angelica sinensis *(Oliv.)	Porcine chondrocytes	↓IL-1*β*, ↓TNF-*α*, ↓MMP-1, ↓MMP-13, ↑SOX9	[83]
(14)	Gentiopicroside	*Gentiana macrophylla*	Rat chondrocyte	↓phosphorylation of p38, ERK, and JNK, ↓MMPs, ↑type II collagen,	[69]
(15)	Ginsenoside Rb1	Ginseng	Rat chondrocyte Human OA chondrocytes	↑Bcl-xL/Bax ratio, ↓MPT, ↓caspase-3, ↓NO, ↓iNOS, ↓TNF*α*, ↓IL-1*β*, ↑collagen type II, ↑SOX9, ↓MMP-1, ↓MMP-13, ↓Notch signaling	[38–40, 81]
(16)	Ginsenoside Rg1, Rg3, Rg5, Rk1, Rf, Rd, Rc, F4	Ginseng	Rat chondrocyte Human OA chondrocytes	↑Bcl-xL/Bax ratio, ↑TIMP-1, ↓cytochome* c*, ↓MMP-13, ↓Bax, ↓Bad, ↓p53, ↓COX-2, ↓NF-*κ*B signaling	[41–44]
(17)	Honokiol	*Magnolia officinalis*	Human OA chondrocytes	↓iNOS, ↓COX-2, ↓NO, ↓PGE_2_, ↓IL-6, ↓MMP-13, ↓NF-*κ*B signaling	[15]
(18)	Icariin	*Epimedium pubescens*	Rabbit chondrocytes SW 1353	↑SOX9, ↑collagen type II, ↑aggrecan, ↓MMP-13, ↓NF-*κ*B signaling, ↓phosphorylations of p38, JNK, *β*-catenin,	[78–80]
(19)	Monotropein	*Morinda officinalis How*	Rat chondrocyte	↓MMP-3, ↓MMP-13, ↑COL2A1	[65]
(20)	Morin	*Moraceae*	Rat in vivo and in vitro	↓phosphorylation of p38 and ERK1/2, ↓MMP-3, ↓MMP-13, ↑TIMP-1	[75]
(21)	Nicotine	Cigarette	Mouseand rat in vivo, Rat chondrocyte	↑PI3K/Akt/Bcl-2 signaling, ↑nAChR, ↓MMP-13, ↑TIMP-1, ↓IGF-1 signaling, ↑PI3K/Akt/p70S6K/S6 signaling,	[56, 57, 66, 101]
(22)	Pinocembrin	Propolis	Human OA chondrocytes	↓MMP-3, ↓MMP-13, ↓NF-*κ*B signaling	[82]
(23)	Piperine	*Piper nigrum*	Human OA chondrocytes	↓iNOS, ↓COX-2, ↓NO, ↓PGE_2_, ↓MMPs, ↓NF-*κ*B signaling	[18]
(24)	Protocatechuic acid	Green tea, catechins	Rabbit chondrocytes	↑aggrecan, ↑type II collagen, ↑SOX9	[98]
(25)	Psoralen	*Fructus Psoraleae*	Rat chondrocyte	↑aggrecan, ↑type II collagen, ↑SOX9	[99]
(26)	Resveratrol	Cranberries, peanuts, red grapes, *Polygonum cuspidatum*	Rat chondrocyte Rabbit chondrocytes Human OA chondrocytes	↑SIRT1, ↑collagen type II, ↓IL-1*β*, ↓TNF*α*, ↓PGE_2_, ↓NO, ↓iNOS, ↓COX-2, ↓NF-*κ*B signaling, ↓TLR4, ↓MyD88, ↓TRAF6, ↓MMP-13, ↓NF-*κ*B and AP-1 signaling, ↑MAPK signaling, ↓HIF-2*α*	[24–26, 35, 36, 87, 88, 109]
(27)	Rutin	—	Pig in vivo	↓Coll2-1, ↓Fib3-1, ↓Fib3-2, ↓Coll2-1NO2	[91]
(28)	Saponin	*Clematis chinensis*	Rat in vivo	↓degradation of ECM	[92]
(29)	Schisandrae Fructus	*Schisandra chinensis*	SW1353	↓MMP-1, ↓MMP-3, ↓MMP-13, ↓COX-2, ↓iNOS, ↓phosphorylation of p38, ERK1/2, and JNK, ↓NF-*κ*B signaling	[74]
(30)	Sinomenine	*Sinomenium acutum*	rabbit chondrocyte	↓release of GAG, ↓MMP-13, ↓caspase 3, ↑TIMP-1	[64]
(31)	Tetramethylpyrazine	*Ligusticum wallichii Franchat*	Rabbit chondrocyte	↓ROS, ↓caspase-3, ↓MMP-3, ↓MMP-13, ↓COX-2, ↓iNOS, ↓type X collagen, ↑TIMP-1, ↑type II collagen	[52, 62]
(32)	Tetrandrine	*Stephania tetrandra *S. Moore	RAW264.7 cell, ATDC5 cells	↓IL-6, ↓IL-1*β*, ↓TNF*α*, ↓PGE_2_, ↓NO, ↓MMPs, ↓NF-*κ*B signaling, ↓*β*-catenin signaling	[22] [84]
(33)	TMF	*Murraya exotica*	Rat chondrocyte	↓EP/cAMP/PKA, ↓*β*-catenin signaling, ↓PERK, ↓ATF6, and ↓IRE1 signaling	[54, 55]
(34)	Withaferin A	*Withania somnifera*	Rabbit chondrocytes	↑collagen type II, ↓COX-2, ↓PI3K/Akt, ↓p38, ↓JNK signaling	[37]
(35)	—	*Bauhinia championii*	Rat chondrocyte	↑Wnt/*β*-catenin signaling, ↑Frizzled-2, ↑Wnt-4, ↑*β*-catenin, ↑Cyclin D1, ↓GSK-3*β*	[96]
(36)	—	Bushen Zhuangjin Decoction	Rat chondrocyte	↓BIP, ↓ATF4, ↓CHOP, ↓caspase-9, ↓caspase-3, ↓Bax, ↑XBP1, ↑Bcl-2 ↑CDK4, ↑CDK6, ↑Cyclin D1, ↑G1/S cell cycle transition	[53] [94]
(37)	—	Duhuo Jisheng Decoction	Rat chondrocyte	↑Bcl-2, ↓Bax, ↓caspase-3, ↓caspase-9, ↑CDK4, ↑CDK6, ↑Cyclin D1, ↑G1/S cell cycle transition, ↓VEGF, ↓HIF-1*α*	[50] [95, 104]
(38)	—	*Eucommiaulmoides*	Rat in vivo	↓MMP-1, ↓MMP-3, ↓MMP-13, ↓PI3K/Akt signaling	[76, 77]
(39)	—	Fuyuan Decoction	SW1353 cells	↓iNOS, ↓NO,↓NF-*κ*B signaling, ↑phosphorylation of Smad2/3, ↑COL2A1, ↑SOX9	[51, 97]
(40)	—	Guilu Erxian Jiao	Humanin vivo	↑muscle strength, ↓joint pain, ↓Lequesne index scores	[105]
(41)	—	Juanbi capsule	Rabbit in vivo	↓MMP-2, ↓MMP-9	[63]
(42)	—	*Murraya exotica*	Rat chondrocyte	↓*β*-catenin, ↓COX-2, ↓TNF*α*, ↓IL-*β*	[110]
(43)	—	*Phellodendron amurense*	Human OA chondrocytes	↑type II collagen, ↑TIMP-1, ↓MMP, ↓p-ERK1/2, JNK, and p38 MAPK,	[68]
(44)	—	Sanmiao formula	Rat chondrocyte Rat in vivo	↓MMP-3, ↓ADAMTS-4, ↑TIMP-1, ↑TIMP-3	[59]
(45)	—	Tougu Xiaotong	UMR-106	↑Proliferation, ↓cell mortality, ↓inflammatory cytokine	[106]
